# Attachment style, dyadic adjustment and gender roles attitudes of trans men and their partners in Turkey

**DOI:** 10.1192/j.eurpsy.2025.2389

**Published:** 2025-08-26

**Authors:** M. Az, N. Direk, B. E. Onur Aysevener

**Affiliations:** 1 Department of Psychiatry, Private Office, İzmir; 2 Department of Psychiatry, Istanbul Medical Faculty, Istanbul, Türkiye

## Abstract

**Introduction:**

It is being thought that the presence and quality of relationships to psychosocial adjustment and well-being is important. Little is known about the romantic relationships of individuals diagnosed with gender dysphoria. In the last thirty years, attachment theory has become one of the main references for adult romantic relationship studies. Despite the importance of attributed to the attachment system for overall psychological well-being and the quality of adult relationships, little research has focused on the attachment of individuals with gender dysphoria

**Objectives:**

The present study focuses onresearching the attachment style, dyadic adjustment and gender roles attitudes in trans men and their partners.

**Methods:**

60 trans men and their 50 partners are included in this study.”Experiences in Close Relationships-Revised”, “Gender Roles Attitude Scale”,”Childhood Trauma Questionnaire”, “Dyadic Adjustment Scale”, “Beck Depression Inventory” and “Beck AnxietyInventory” are conducted to all attendees. Also, we used data form which is included sociodemographic and family features for all participants

**Results:**

**Prevalence of insecure attachment style was found high in both groups. Regarding the correlation between attachment style and dyadic adjustment, the dyadic adjustment points in trans men with secure attachment style were found significantly higher than trans men with insecure attachment style (Table 2,p:0,006). The average relationship duration of the participants with secure attachment style was higher than the participants with insecure attachment style. It was found that both groups have egalitarian attitudes in general terms. The egalitarian attitudes subscale points of gender role attitude scale in trans men were found significantly higher than the egalitarian attitudes subscale points of gender role attitude scale in partners (p:0,025)
**

**Image 1:**

**Figure:**
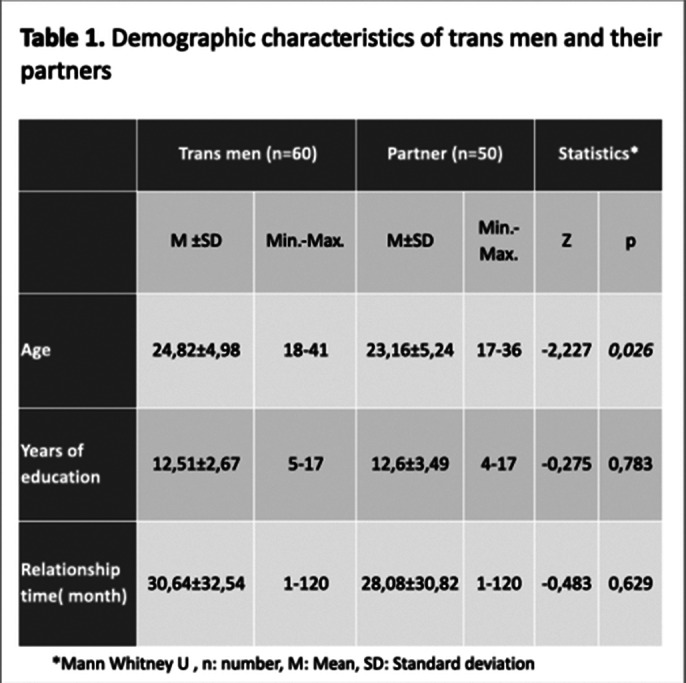

**Image 2:**

**Figure:**
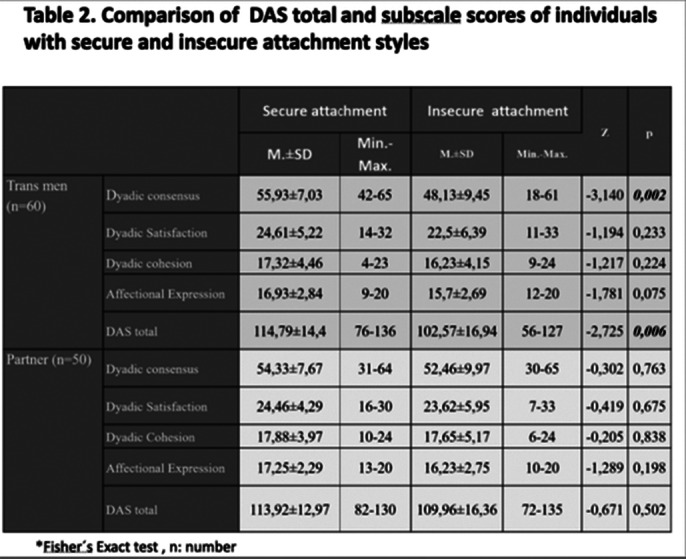

**Conclusions:**

The present study constitutes the first study in Turkey, which researching the attachment style, dyadic adjustment and gender roles attitudes in trans men and their partners, was analyzed. There are few studies in the literature investigating the gender roles of trans men and their partners. In this study, insecure attachment rates were found to be high in both groups. Attachment styles and romantic relationships are fundamental components for psychosocial adjustment and well-being. For this reason, attachment styles should be made a part of the clinical evaluation of transgender people. It should be aimed to increase the sense of security of the person. Thus, clinical studies that should evaluate attachment styles as part of a standardized assessment and increase one’s sense of security must be produced primarily. Therefore, individual and group psychotherapeutic work aimed at reshaping internal working models could directly or indirectly facilitate access to support groups, reinforce a more positive self-image.

**Disclosure of Interest:**

None Declared

